# Generation of Cas9 transgenic zebrafish and their application in establishing an ERV-deficient animal model

**DOI:** 10.1007/s10529-018-2605-5

**Published:** 2018-09-22

**Authors:** Zhe Yang, Shihao Chen, Songlei Xue, Xinxiu Li, Zhen Sun, Yu Yang, Xuming Hu, Tuoyu Geng, Hengmi Cui

**Affiliations:** 1 Institute of Epigenetics and Epigenomics, Yangzhou University, Yangzhou, Jiangsu 225009 China; 2 College of Animal Science and Technology, Yangzhou University, Yangzhou, Jiangsu 225009 China; 3 Institute of Comparative Medicine, Yangzhou University, Yangzhou, Jiangsu 225009 China; 4 Jiangsu Co-innovation Center for Prevention and Control of Important Animal Infectious Diseases and Zoonoses, Yangzhou University, Yangzhou, Jiangsu 225009 China; 5 Joint International Research Laboratory of Agricultural & Agri-Product Safety, The Ministry of Education of China, Yangzhou University, Yangzhou, Jiangsu 225009 China; 6 College of Animal Science and Technology, Yangzhou University, Yangzhou, Jiangsu 225000 China

**Keywords:** CRISPR/Cas9, Zebrafish, Spinal abnormality, Embryonic development, Genomic editing

## Abstract

**Objectives:**

To investigate the effect of endogenous Cas9 on genome editing efficiency in transgenic zebrafish.

**Results:**

Here we have constructed a transgenic zebrafish strain that can be screened by pigment deficiency. Compared with the traditional CRISPR injection method, the transgenic zebrafish can improve the efficiency of genome editing significantly. At the same time, we first observed that the phenotype of vertebral malformation in early embryonic development of zebrafish after ZFERV knockout.

**Conclusions:**

The transgenic zebrafish with expressed Cas9, is more efficient in genome editing. And the results of ZFERV knockout indicated that ERV may affect the vertebral development by Notch1/Delta D signal pathway.

**Electronic supplementary material:**

The online version of this article (10.1007/s10529-018-2605-5) contains supplementary material, which is available to authorized users.

## Introduction

Zebrafish are a classical animal model for developmental biology and biomedical studies; several important questions in vertebrate development have been addressed using this model. Particularly, large-scale screening in the 1990s for zebrafish mutants generated via random mutagenesis resulted in zebrafish becoming one of the most frequently used animal models.

In recent years, newly developed genome-editing tools, including ZFNs, TALENs, and CRISPR/Cas9, provide a feasible approach for the generation of zebrafish with precise genomic modifications. These zebrafish have significantly promoted the understanding of genes with unknown functions, especially those involved in embryonic development (Jao et al. 2013). Specifically, CRISPR/Cas9 has become a popular method for site-specific genome editing due to its convenience and efficiency. However, as fertilized zebrafish eggs develop quickly, direct delivery of large-sized Cas9 mRNA or protein into eggs frequently poses the problem of chimerism. Generally, establishing a zebrafish homozygous line with a genomic modification from the mosaic F0 generation of zebrafish is time consuming, requiring breeding and genotyping of the F1 and F2 generations (Varshney et al. 2015). Thus, generating homozygous zebrafish with genomic modifications at the F0 generation would be very efficient and useful. Transgenic Cas9 zebrafish may provide an opportunity to meet this requirement.

In addition, transgenic Cas9 zebrafish may facilitate the generation of zebrafish with the deletion of a large genomic fragment. The CRISPR/Cas9 system has been used to knock out long non-coding RNAs that are usually large in size (Wen et al. 2016), as well as other large genomic DNA sequences (Kraft et al. 2015). Endogenous retroviruses (ERVs), which might be remnants of ancient retroviral infections, are large genomic elements. Zebrafish possess an 11349-bp ERV on chromosome 19 that contains the *Gag, Pol*, and *Env* genes, as well as long terminal repeat (LTR) sequences (Shi et al. 2015). Previous studies have shown that ERVs play an important role in the activation of innate immunity, embryonic development and the occurrence of diseases and tumors (Chuong et al. 2016). Given that zebrafish embryos can develop in vitro and because the effects of ERV deletion can be observed during embryonic development, ERV-deficient zebrafish may serve as a good model for ERV studies.

In this study, a Cas9 transgenic zebrafish line with a selective marker for homozygosity was successfully established, solving the problem of chimerism that occurs following the injection of large-sized Cas9 mRNA or protein into fertilized zebrafish eggs. Moreover, the generation of ERV-deficient zebrafish using the Cas9 transgenic zebrafish line indicates that the transgenic Cas9 gene is active and can be used to modify any gene of interest in zebrafish. Finally, ERV-deficient zebrafish may serve as animal models for ERV studies, and the spinal abnormality observed in ERV-deficient fish was associated with the downregulation of the delta D and Notch 1 genes.

## Materials and methods

### Maintenance and breeding of zebrafish

TU wild-type zebrafish (*Danio rerio*) were obtained from China Zebrafish Resource Center or CZRC (Wuhan, China). The fish were maintained at 28.5 °C under a 14 h light: 10 h dark–light regime (i.e., 14hL: 10hD) in the Zebrafish Research Facility, Yangzhou University (Yangzhou, China). To generate offspring, mating was carried out at a ratio of 1 female to 2 males, followed by natural spawning. Embryos were raised in E3 medium (5 mmol/L NaCl, 0.17 mmol/L KCl, 0.33 mmol/L CaCl_2_, and 0.33 mmol/L MgSO_4_, pH 7.2) at 28.5 °C on a 14 h light:10 h dark cycle. All animal experiments were performed in accordance with the guidelines for animal welfare in China, and the animal protocol was approved by the animal welfare committee of Yangzhou University.

### Acquisition of zebrafish endogenous promoters and construction of eGFP expression plasmids

The zebrafish *Eef1g* (eukaryotic translation elongation factor 1 gamma) (Burket et al. 2008) and *Ef1α* (elongation factor 1) (Moon et al. 2013) promoter sequences were PCR-amplified using the primers specific to the promoters (Supplemental Table 1) and genomic DNA samples isolated from zebrafish. To link the promoters to the eGFP expression plasmid, the promoter sequences were further PCR-amplified using primers containing MluI (New England Biolabs (NEB), USA, Catalog no. R3198S) and NheI (NEB, USA, Catalog no. R3131S) restriction sites. The eGFP fragments were obtained by amplifying the pIRES2-eGFP plasmid using the primers eGFP-BamHI-F and eGFP-XhoI-R (Supplemental Table 1). The fragment was inserted into the pcDNA3.1 plasmid by digesting the plasmids with BamHI (NEB, USA, Catalog no. R3136S) and XhoI (NEB, USA, Catalog no. R0146S), followed by ligation with T4 ligase according to the manufacturers’ instructions. The map of the eGFP expression vector is shown in Supplemental Fig. 1. To generate eGFP constructs driven by the zebrafish *Eef1g* or *Ef1α* promoter, the CMV promoter was removed from the pcDNA3.1-eGFP plasmid by digestion with MluI and NheI (Supplemental Fig. 2), followed by ligation with the zebrafish *Eef1g* or *Ef1a* promoter using T4 ligase according to the manufacturers’ instructions. The resulting pcDNA3.1 plasmids containing *Ef1α*-eGFP or *Eef1g*-eGFP fragments are shown in Supplemental Figs. 3 and 4.

### Microinjection of eGFP expression cassettes driven by zebrafish promoters

To generate zebrafish with *Eef1g*-eGFP or *Ef1α*-eGFP expression cassettes, microinjection solutions were prepared containing 100 ng *Eef1g*-eGFP (i.e., pcDNA3.1 [*Eef1g*-eGFP]) or *Ef1α*-eGFP (i.e., pcDNA3.1 [*Ef1α*-eGFP]) plasmid, 1% phenol red indicator, and water up to 5 µL (Supplemental Fig. 1). pcDNA3.1 [eGFP] plasmid containing eGFP and no promoter was used as a control (Supplemental Fig. 1). Microinjection was performed using a microinjector (TRITECH™ RESEARCH microINJECTOR system, Veteran Avenue, Los Angeles, USA, Catalog no. MINJ-1) under an inverted microscope (Zebrasc, Beijing, China) as described previously (Rosen et al. 2009). Each fertilized zebrafish egg was injected with 1 nL of each solution at the one or two-cell stage, and the injected embryos were subsequently raised at 28 °C in E3 medium. The fluorescent signal from the injected embryos was examined on a fluorescence microscope (Olympus FluoView IX73, Japan, Catalog No. IX7301F). The promoter activity was evaluated based on fluorescence emission.

### Construction of Cas9 expression cassettes

The DNA sequence of the Cas9 gene was synthesized from the MLM3613 plasmid (Addgene, Catalog no. 42251). For the construction of the Cas9 expression cassette, the *Ef1α* promoter was inserted into MLM3613 plasmid via Sep I (NEB, Catalog No. R3133S) and NheI digestion, followed by ligation with T4 ligase according to the manufacturers’ instructions. The expression cassette consists of the following elements: *Ef1α*-Cas9-NLS-bGH poly (A). For locus-specific integration of the Cas9 expression cassette at the *Mitfa* locus in the zebrafish genome, Genewiz, Inc. (Suzhou, China), synthesized the 40-bp-long homologous recombination arms (HRL and HRR) around the *Mitfα* sgRNA identification site, together with the linker primers Cas9 cassette-F and Cas9 cassette-R (Supplemental Table 1). The purified product was used in PCR with the aforementioned Cas9 expression cassette to produce the Cas9 transgenic cassette: HRL-*Ef1α*-Cas9-NLS-bGH poly (A)-HRR. The PCR procedure included initial denaturation at 92 °C for 2 min, followed by 30 cycles of 92 °C for 30 s, 62 °C for 2 min 30 s, and 72 °C for 10 min, which was based on Vazyme’s long chain PCR settings (Vazyme, China, Catalog no. P501-d1). The final PCR product was isolated and purified using the Gel Recovery Kit (OMEGA, USA, Catalog no. D2500-02) according to the manufacturer’s instructions.

### In vitro sgRNA and Cas9 mRNA synthesis

sgRNAs for *Mitfα*, *Tyr* and zebrafish ERV (*ZFERV*) were designed by Chop–Chop (Labun et al. 2016). The DNA templates for in vitro sgRNA synthesis, which contained the T7 promoter, a 20-nucleotide (nt) long guide sequence, and an sgRNA scaffold (Chen et al. 2013), were generated by overlapping PCR (Lin et al. 2014). PCR was performed using Phanta™^®^ Super-Fidelity DNA polymerase (Vazyme, Catalog no. P501-d1) and the annealed product of two oligos (Supplemental Table 1) (Varshney et al. 2015). The annealing and PCR reactions contained 5 µL of 5× Super-Fidelity (SF) Buffer, 1 µL of dNTP Mix, 1 µL each of 10 µM oligos, 1 µL of Phanta^®^ Super-Fidelity DNA Polymerase and 16 µL of water. The PCR settings were the same as those described previously (Chen et al. 2016). The sgRNA sequences are shown in Table 2. In vitro sgRNA transcription was carried out in reactions containing 1 µg of DNA template, 2 µL of 10× reaction buffer, 2 µL of ATP (100 mM), 2 µL of GTP (100 mM), 2 µL of UTP (100 mM), 2 µL of CTP (100 mM), 2 µL of T7 RNA Polymerase Mix (HiScribe™ T7 High Yield RNA Synthesis Kit, NEB, Catalog no. E2040S), and water up to 20 µL. In vitro transcription was performed by incubation at 37 °C for more than 16 h, followed by treatment with RNase-free DNase I (NEB, Catalog no. M0303S) at room temperature for 30 min. The synthesized sgRNAs were purified by the phenol–chloroform method and dissolved in pure water. To acquire Cas9 mRNA, Cas9 DNA templates from the MLM3613 plasmids were used for in vitro transcription. The templates were acquired by digesting the MLM3613 plasmid with PmeI (NEB, Catalog no. R0560S) and were purified using the Cycle Pure Kit (OMEGA, Catalog no. D6492-02) according to the manufacturers’ instructions. In vitro transcription reactions contained 1 µg of DNA template, 10 µL of 2× NTP/CAP, 2 µL of 10× reaction buffer, 2 µL of T7 RNA polymerase (mMESSAGE mMACHINE*™* T7 Transcription Kit, Thermo Fisher, Catalog no. AM1344), and water up to 20 µL. The settings for in vitro transcription included incubation at 37 °C overnight, followed by treatment with RNase-free DNase I at room temperature for 30 min. The synthesized Cas9 mRNAs were purified by the phenol–chloroform method and dissolved in pure water. All synthesized RNA products were stored at -80 °C before microinjection.

### Generation of Cas9 transgenic zebrafish

To generate zebrafish containing the Cas9 expression cassette, microinjection solutions were prepared containing 1000 ng of Cas9 mRNA, 200 ng of *Mitfα* sgRNA, 500 ng of Cas9 donor fragment, 1% phenol red, and water up to 5 µL. Fertilized zebrafish eggs were injected with 1 nL of solution, and embryos were raised at 28 °C in E3 medium.

### Genotyping and sequencing analysis of transgenic zebrafish

For genotyping, genomic DNA was extracted from the caudal fins of 1-month-old fries, as described previously (Liu and Zhang 2011). Genotyping was performed by PCR using the gene-specific primers Cas9-test-F and Cas9-test-R (Supplemental Table 1), followed by sequencing analysis. To validate the integration of the Cas9 gene at the *Mitfα* locus, PCR was performed with a pair of primers that recognize Cas9 or *Mitfα* gene sequences. The 50-µL PCR reaction contained 10 µL of 5× SF Buffer, 1 µL of dNTP Mix, 2 µL of Cas9 insertion-test-F, 2 µL of Cas9 insertion-test-R, 1 µL of Phanta^®^ Super-Fidelity DNA Polymerase, and 34 µL of water. The PCR settings included denaturation at 94 °C for 5 min, followed by 30 cycles of 94 °C for 30 s, 60 °C for 30 s, and 72 °C for 1 min, and 72 °C for 10 min for final extension. The purified PCR product was subjected to sequencing analysis by Genewiz, Inc.

### RT-PCR-based evaluation of transgenic Cas9 gene expression in the F2 generation

To determine the expression of the transgenic Cas9 gene, the total RNA was isolated from F2 zebrafish embryos with the Nacre phenotype using TRIzol (Ambion, Catalog no.15596018) according to the manufacturer’s instructions. Complementary DNA was synthesized using the total RNA samples and the PrimeScript™ 1st Strand cDNA Synthesis Kit (TAKARA, Catalog no.6110B) according to the manufacturer’s instructions. PCR was subsequently conducted with the cDNA samples and the primers Cas9-test-F and Cas9-test-R (Supplemental Table 1). Additionally, mock reverse transcription with the total RNA was performed as a negative control (no reverse transcriptase added), while PCR using the genomic DNA isolated from the F2 zebrafish was used as a positive control. All PCR products were separated via 1% agarose gel electrophoresis.

### Generation of zebrafish with a *Tyr* gene mutation using Cas9 mRNA or transgenic Cas9

To generate *Tyr*-mutant zebrafish, two solutions for microinjection were prepared. The first solution, injected into Cas9 transgenic fish, contained 200 ng of *Tyr* sgRNA, 1% phenol red, and water up to 5 µL. The second solution, injected into wide-type TU fish, contained 1000 ng of Cas9 mRNA, 200 ng of *Tyr* sgRNA, 1% phenol red, and water up to 5 µL. Embryos were injected with 1 nL of solution. All embryos were maintained at 28 °C for at least one day before viability evaluation.

### T7E1 mutagenesis assay of the *Tyr* gene

The T7E1 assay was performed as described previously (Kim et al. 2009). Genomic DNA was isolated from control TU zebrafish, those developed from Cas9 transgenic zebrafish fertilized eggs injected with *Tyr* sgRNA, and those developed from TU zebrafish fertilized eggs injected with Cas9 mRNA and *Tyr* sgRNA. A general PCR was performed with the genomic DNA samples and the primers *Tyr*-test-F and *Tyr*-test-R (Supplemental Table 1). After gel purification, 200 ng of purified DNA was reannealed in a thermal cycler under the following conditions: 95 °C for 2 min, 95 °C to 85 °C at a rate of 2 °C/s and 85 °C to 25 °C at a rate of 0.1 °C/s. Next, 16 µL of the reannealed product was incubated in a reaction solution containing 0.2 µL of T7 endonuclease I (NEB, USA, Catalog no. M0302S), 2 µL of NEB buffer 2, and 1.8 µL of nuclease-free water at 37 °C for 40 min. The digestion product was separated via 1.2% agarose gel electrophoresis.

### Generation of ZFERV-deficient zebrafish

To generate ZFERV-deficient zebrafish and to compare the efficiency of transgenic Cas9 and Cas9 mRNA for genome editing, two microinjection solutions were prepared. One, injected into Cas9 transgenic fish, contained 200 ng of LTR sgRNA, 1% phenol red, and water up to 5 µL. The second, injected into TU fish, contained 1000 ng of Cas9 mRNA, 200 ng of LTR sgRNA, 1% phenol red, and water up to 5 µL. A total of 1 nL of each solution was used for microinjection. All embryos were raised at 28 °C in the E3 medium.

### Detection of ZFERV deficiency

To confirm the ZFERV deletion, genomic DNA was extracted from zebrafish embryos after 24 h of development, from embryos with spinal deformity after 48 h of development, and from embryos with spinal deformity after 120 h of development. PCR was performed with the genomic DNA samples and each of the following primers pairs: for detection of the ZFERV 5′-LTR sequence, 5′LTR-test-F and 5′LTR-test-R (Supplemental Table 1); for detection of the ZFERV full-length sequence, 5′LTR-test-F and 3′LTR-test-R (Supplemental Table 1). The PCR solution contained 2 µL of genomic DNA, 2 µL of primers (1 µL each), 1 µL of Phanta^®^ Super-Fidelity DNA Polymerase, 4 µL of 5× SF Buffer, 1 µL of dNTPs, and 10 µL of water. The PCR settings included denaturation at 94 °C for 10 min, followed by 30 cycles of 94 °C for 30 s, 60 °C for 30 s, and 72 °C for 30 s, and 72 °C for 5 min for final extension. The PCR product was separated via 1% agarose gel electrophoresis and subjected to sequencing analysis by Genewiz, Inc.

### qPCR analysis with the ZFERV-deficient zebrafishes

To determine Env, Delta D, Notch 1 and Her1 gene expression, total RNA was isolated using TRIzol according to the manufacturer’s instruction. Quantitative PCR was performed on a CFX Connect™ Real-time System (Bio-Rad, Serial no. 788BR04566). The reaction solutions contained 10 µL of 2× SYBR^®^Premix ExTaq (Clontech, Japan, Catalog no. 639676), 0.8 µL of Forward Primer (10 µm), 0.8 µL of Reverse Primer (10 µm), 2 µL of cDNA, and water up to 20 µL. The qPCR settings included denaturation at 95 °C for 3 min, followed by 40 cycles of 95 °C for 10 s and 58 °C for 30 s. The primers for the four genes and internal control genes *Gapdh* and *Ef1α* are listed in Supplemental Table 1. Relative gene expression levels were calculated with the 2^−∆∆CT^ method, as indicated in the manufacturer guidelines (Bio-Rad).

### Statistical analysis

One-way analysis of variance (ANOVA) was performed to determine the statistical significance of the difference in the expression of a given gene among different treatments. The level of statistical significance was set at *p* value < 0.05. All data are presented as the mean ± SE.

## Results and discussion

### Selection of the promoter to drive expression of the transgene in zebrafish

**Fig. 1:**
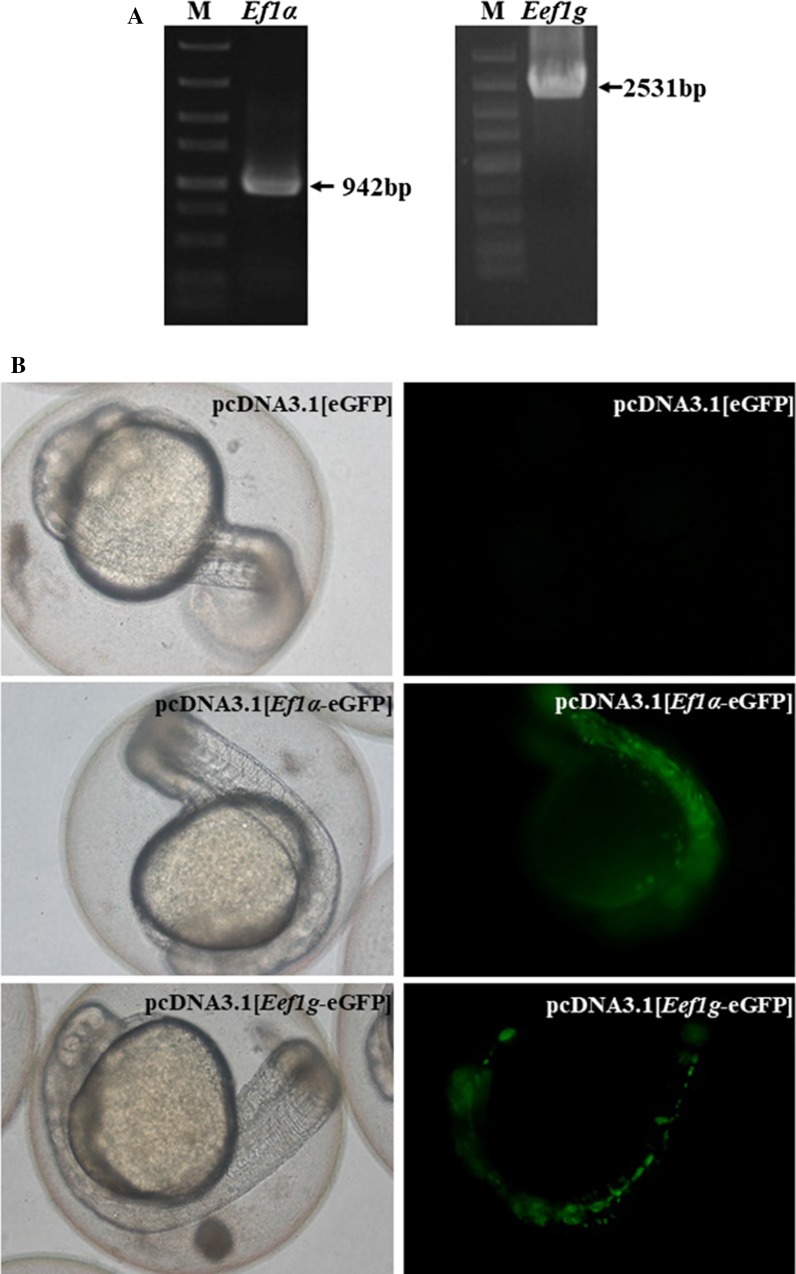
Selection of a promoter to drive transgenic Cas9 expression. **a** Amplification of *Ef1α* (942 bp) and *Eef1g* (2531 bp) promoter sequences. **b** Zebrafish embryos injected with pcDNA3.1 [eGFP], pcDNA3.1 [*Ef1α*-eGFP], and pcDNA3.1 [*Eef1g*-eGFP]. eGFP expression levels were indicated by fluorescent intensity

To efficiently and constantly drive the expression of exogenous Cas9 gene in zebrafish, two candidate endogenous zebrafish promoters, *Eef1g* (eukaryotic translation elongation factor 1 gamma) and *Ef1α* (elongation factor 1), were used in the transgenic study. Both promoters are known to drive genes expressed during early zebrafish development. Using zebrafish genomic DNA as a PCR template, *Ef1α* (942 bp) and *Eef1g* promoter sequences (2531 bp) were successfully amplified (Fig. 1a) and confirmed by sequence analysis of the PCR products. The promoter sequences were separately cloned into expression vectors to drive the expression of a green fluorescent protein reporter gene (Fig. 1b). In total, 482 of 642 zebrafish (76%) in the *Ef1α* group were fluorescent, while 400 of 540 zebrafish (74%) in the *Eef1g* group were fluorescent, suggesting that the two promoters were able to drive reporter gene expression with similarly efficiency. The *Ef1α* promoter is much shorter than the *Eef1g* promoter and may also possess better integration efficiency; we thus chose the *Ef1α* promoter as the promoter for the Cas9 gene to construct the transgenic donor fragment.

### Identification of Cas9 transgenic zebrafish

**Fig. 2:**
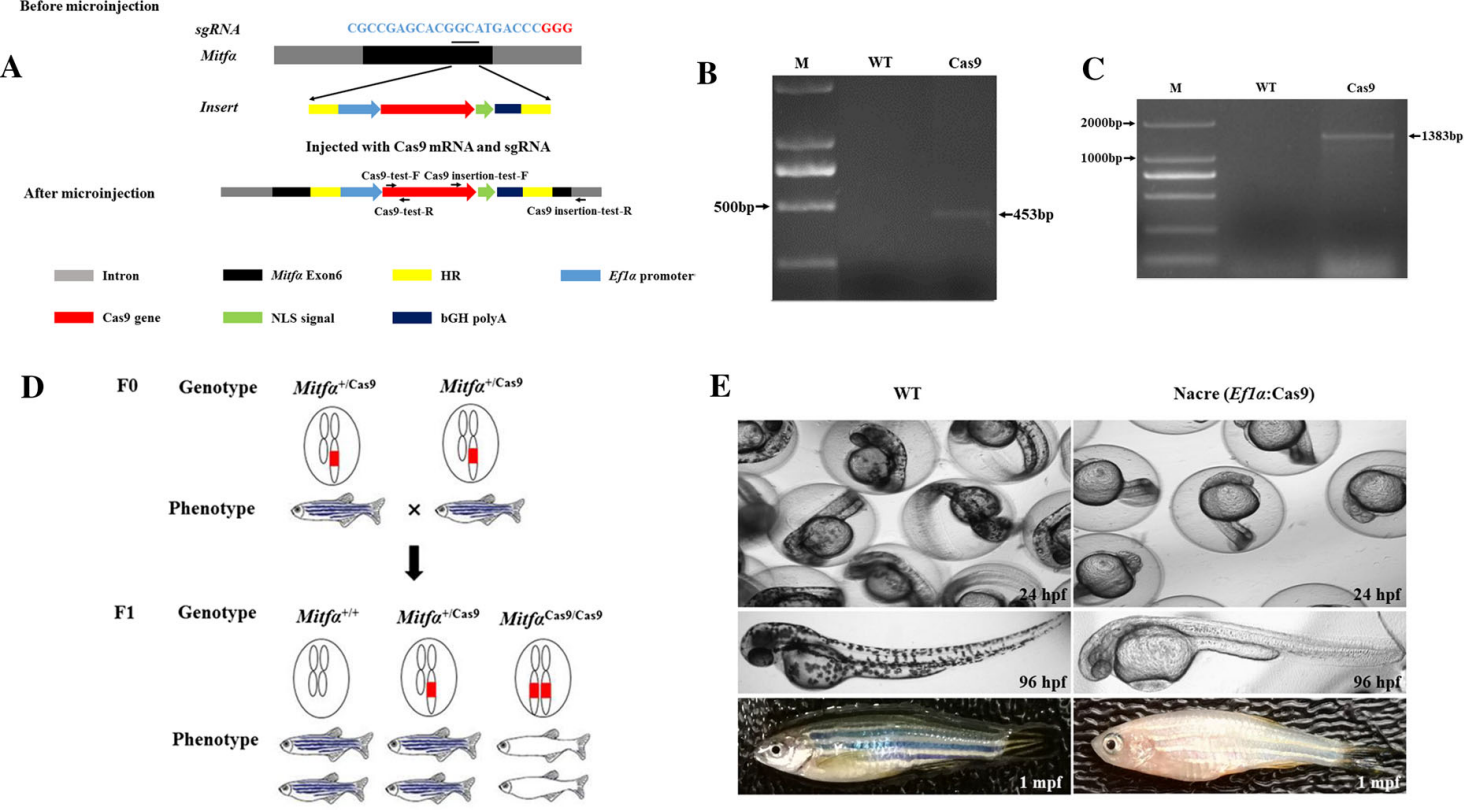
Generation of Cas9 transgenic zebrafish. **a** Schematic illustration of *Mitfα* locus-specific integration of the *Ef1α*-Cas9 expression cassette based on homologous recombination using Cas9 mRNA and sgRNA targeted to the zebrafish *Mitfα* gene. The indicated primers were used to confirm integration. HR denotes homologous recombination arm, and NLS denotes nuclear localization signal. **b** Confirmation of Cas9 gene insertion into the zebrafish genome, indicated by amplification of Cas9 by PCR with Cas9-specific primers. The expected size of the Cas9 amplicons is 453 bp. TU denotes genomic DNA isolated from wild-type zebrafish, while Cas9 denotes genomic DNA isolated from Cas9 transgenic zebrafish. M denotes the DNA ladder. **c** Confirmation of *Mitfα*-locus-specific integration of the Cas9 gene into the zebrafish genome, indicated by amplification of Cas9 by PCR with both *Mitfα*- and Cas9-specific primers. The expected size of the Cas9 amplicons is 1383 bp. TU denotes genomic DNA isolated from wild-type zebrafish, while Cas9 denotes genomic DNA isolated from Cas9 transgenic zebrafish. M denotes the DNA ladder. **d** Selection of zebrafish with biallelic transgenic Cas9 gene based on the nacre phenotype (pigment-deficient mutant), including genotyping and breeding of F0 zebrafish and selection for nacre phenotype-carrying zebrafish in F1. Based on phenotype, 23% (260/1130) of F1 zebrafish carry the nacre phenotype. The red box denotes the transgenic Cas9 gene. **e** Comparison of phenotypes of TU wild-type zebrafish with those of *Mitfα*-locus-specific transgenic Cas9 zebrafish at different developmental stages. Hpf and mpf indicate hours post-fertilization and months post-fertilization, respectively

**Fig. 3:**
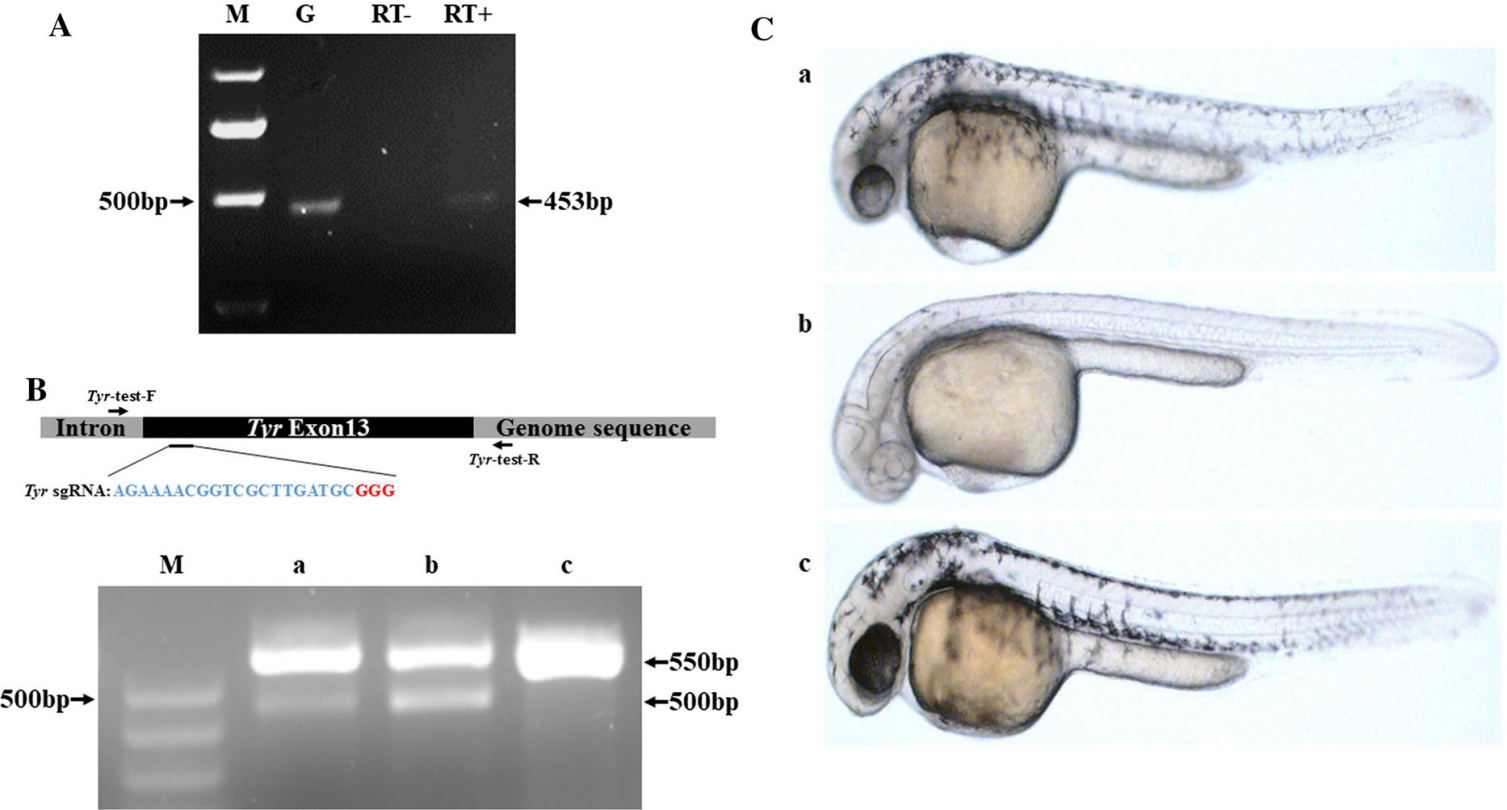
Validation of Cas9 activity in F2 transgenic zebrafish. **a** Transgenic Cas9 expression was detected by reverse transcription (RT)-PCR in F2 transgenic zebrafish. Lane G: genomic DNA from F2 transgenic zebrafish as the positive control. Lane RT-: RT with no reverse transcriptase as the negative control. Lane RT + : cDNA synthesized by RT with total RNA isolated from F2 transgenic zebrafish. Lane M: DNA ladder. The expected size of the amplicon is 453 bp. **b** Validation of transgenic Cas9 activity by *Tyr* mutation assay and comparison of the editing efficiency of exogenous Cas9 mRNA and transgenic Cas9. The top panel shows the targeting site of the Tyr sgRNA in the Tyr gene, as well as the binding sites of the PCR primers used to detect the *Tyr* mutation in F2 zebrafish. The bottom panel shows the detection of the *Tyr* mutation via PCR and T7E1 digestion in fertilized eggs from TU wild-type zebrafish, which were injected with *Tyr* sgRNA plus Cas9 mRNA (**a**) vs. fertilized eggs from Cas9 transgenic zebrafish, which were injected with *Tyr* sgRNA (**b**), and TU wildtype zebrafish as control (**c**). The *Tyr* mutation was indicated by two fragments (approximately 550 bp and 500 bp, as indicated). **c** Different phenotypic changes in pigmentation fading in *Tyr* knockout zebrafish embryos produced via different gene editing methods. The representative images show **a** embryos injected with Cas9 mRNA and sgRNA, **b** Cas9 transgenic embryos injected with sgRNA and **c** wild-type embryos

Using genomic DNA isolated from the fins of F0 zebrafish developed from injected eggs, Cas9 was PCR-amplified using specific primers, indicating that the Cas9 gene was successfully integrated into the zebrafish genome via the CRISPR-Cas9 system (119/403, approximately 30% of F0 zebrafish). Amplicons with the expected size of 453 bp were sequenced and confirmed (Fig. 2b). To confirm if the integration of the Cas9 gene was correctly targeted to the *Mitfα* locus, a pair of primers (Cas9 insertion-test-F and Cas9 insertion-test-R) was designed to be specific to both the Cas9 gene and the flanking *Mitfα* sequence. A PCR amplicon with of the expected size (1383 bp) was obtained (Fig. 2c), and subsequent sequencing analysis confirmed that the Cas9 gene was specifically inserted into the *Mitfα* locus. To evaluate the activity of the inserted Cas9 gene, zebrafish that developed from the injected eggs were used to generate the next generation of zebrafish with biallelic disruption of the *Mitfα* gene. As only fish with biallelic *Mitfα* disruption would exhibit a distinct phenotype from those with monoallelic or no *Mitfα* disruption in terms of body pigmentation (Fig. 2d), offspring with biallelic-mutation *Mitfα* were easily selected by appearance (260 out of 1130 or 23%). Transgenic Cas9 expression gene was detected in these offspring (Fig. 2e), suggesting that Cas9 was constitutively expressed in the transgenic zebrafish line (Fig. 3a). Using this line, sgRNA targeted to *Tyr* was injected to generate *Tyr*-mutant zebrafishes. T7E1 restriction enzyme digestion showed that *Tyr* was successfully mutated. The results also indicated that the editing efficiency of transgenic Cas9 was better than that of exogenous Cas9 mRNA (Fig. 3b), and the phenotypic change caused by *Tyr* deletion is shown in Fig. 3c. These experiments confirmed that the transgenic Cas9 functioned efficiently.

### ERV deletion causes spinal abnormality in zebrafish

**Fig. 4:**
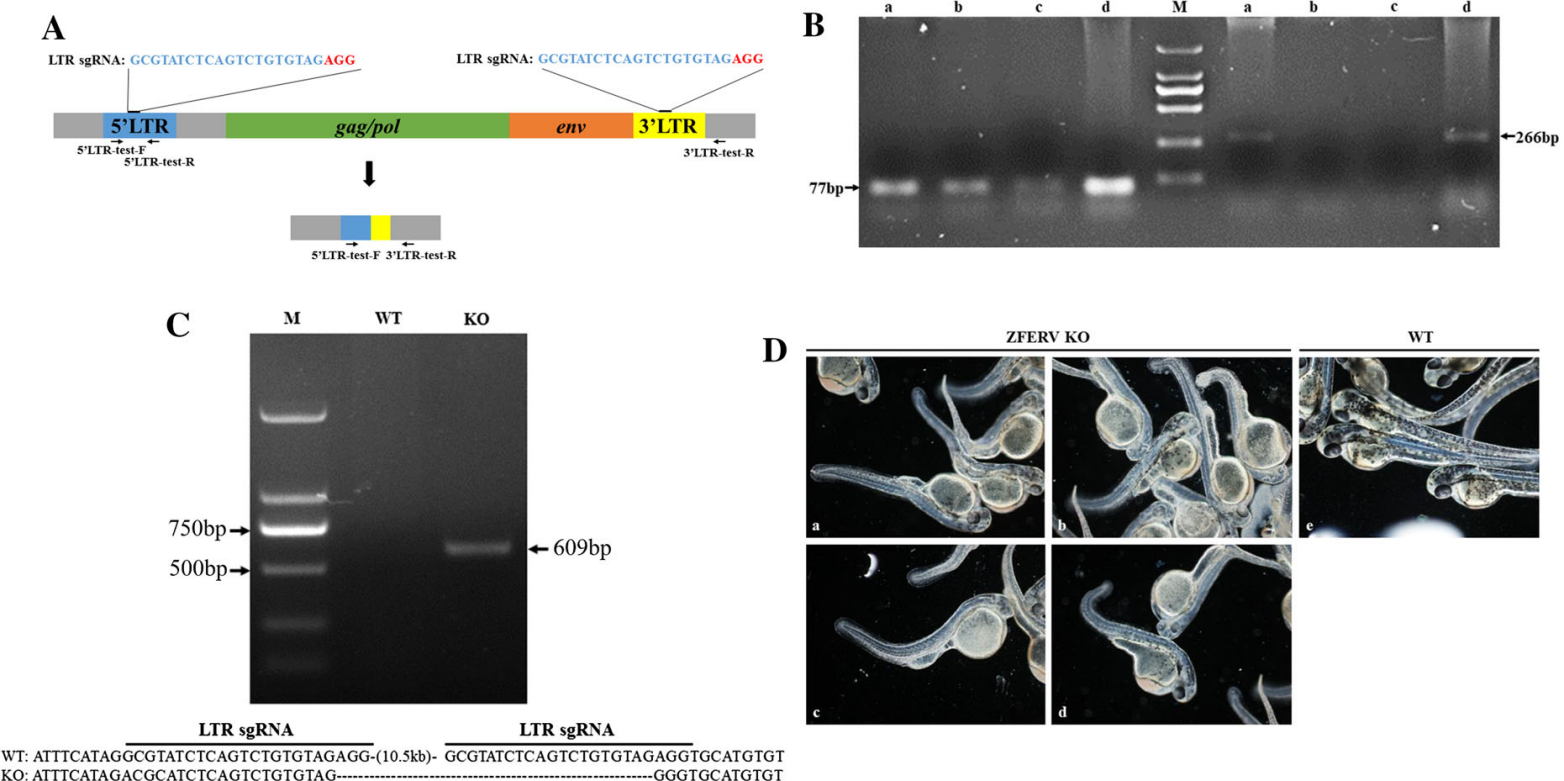
Generation of ERV-deficient zebrafish. **a** Schematic overview of ZFERV gene knockout. 5′LTR-test-F and 5′LTR-test-R primers were used to PCR-amplify the 5′LTR sequence, while 5′LTR-test-F and 3′LTR-test-R primers were used to PCR-amplify the sequence between the 5′ and 3′LTR. **b** Gel images of PCR amplicons synthesized using GAPDH primers and the 5′LTR-test-F and 5′LTR-test-R primers for the ZFERV 5‘LTR used in the KO test. The primers 5′LTR-test-F and 5′LTR-test-R were used to amplify the ZFERV 5′LTR sequence. The expected sizes of the amplicons are indicated. Lane a: collected fertilized zebrafish eggs injected with LTR sgRNAs; Lane b and Lane c: zebrafish embryos with spinal deformity at 48 h and 120 h post-fertilization; Lane d: TU wild-type zebrafish embryos; M denotes the DNA ladder. **c** Detection of ZFERV gene deletion, which is indicated by the gel image of PCR amplicons synthesized from zebrafish genomic DNA using the primers 5′LTR-test-F and 3′LTR-test-R. The primers 5′LTR-test-F and 3′LTR-test-R were used to amplify the region between the 5′ and 3′LTR sequences. The expected size of the amplicons was 609 bp. The amplicon sequences were determined by sequencing analysis and are shown at the bottom. *WT* genomic DNA isolated from TU wild-type zebrafish, *KO* genomic DNA isolated from ERV-knockout zebrafish, *M* DNA ladder. (**d**) Spinal abnormality was caused by various sgRNAs in ZFERV-knockout (KO) zebrafish embryos. ZFERV knockout embryos produced by separately injecting LTR-sgRNA (**a**), sgRNA1 (**b**), sgRNA2 (**c**), or sgRNA3 (**d**) into the Cas9-transgenic zebrafish embryos. **e** Normal spinal morphology in wild-type embryos

Recent studies indicate that ERVs play a role in humans and other animals. As an animal model of ERV knockout is urgently needed, the transgenic Cas9 zebrafish line was used to generate an ERV-knockout model. An sgRNA targeting the long terminal repeat (LTR) of zebrafish ERV (ZFERV, 11349 bp in length) was designed (Fig. 4a). After injection, embryos were collected at different stages, and genomic DNA was then extracted for PCR. The primers 5′LTR-test-F, 5′LTR-test-R were used to amplify the 5′LTR sequence around the sgRNA-targeted site. As expected, no amplicons were observed in the ERV-knockout fish due to the removal of the partial 5′LTR sequence that contains the binding site for the reverse primer (Supplemental Fig. 5), whereas a 284-bp PCR product was detected in wild-type fish (Fig. 4b). Consistently, using the primers 5′LTR-test-F, 3′LTR-test-R, which recognize the 5′LTR and 3′LTR sequences, respectively, a 609-bp amplicon was obtained in the ERV-knockout fish, while the expected 11182-bp fragment from wild-type fish was too large to amplify (Fig. 4c). These results suggested that ERV was successfully knocked out with the Cas9 transgenic zebrafish. Sequencing analysis further confirmed that ERV was removed from the zebrafish. Surprisingly, most of the embryos (2271 out of 2640, or 86%) showed spinal abnormalities 48 h after injection (Fig. 4d). Zebrafish embryos with abnormal spines had difficulty swimming.

To confirm that the abnormality was not caused by off-target effects, three additional sgRNAs target to conserved sequence in 5′LTR and 3′LTR of ZFERV were designed (the sgRNA sequences are shown in Supplemental Table 2) and separately injected into Cas9-transgenic zebrafish embryos. In line with the notion that the spinal abnormality was caused by ZFERV deletion, spinal abnormalities (Fig. 4d) and dramatic reductions in of the Env gene expression (Supplemental Fig. 6) were also observed in embryos treated with these additional sgRNAs. Embryos with spinal abnormalities constituted 84% ~ 89.6% of total embryos (Supplemental Table 3).

**Fig. 5:**
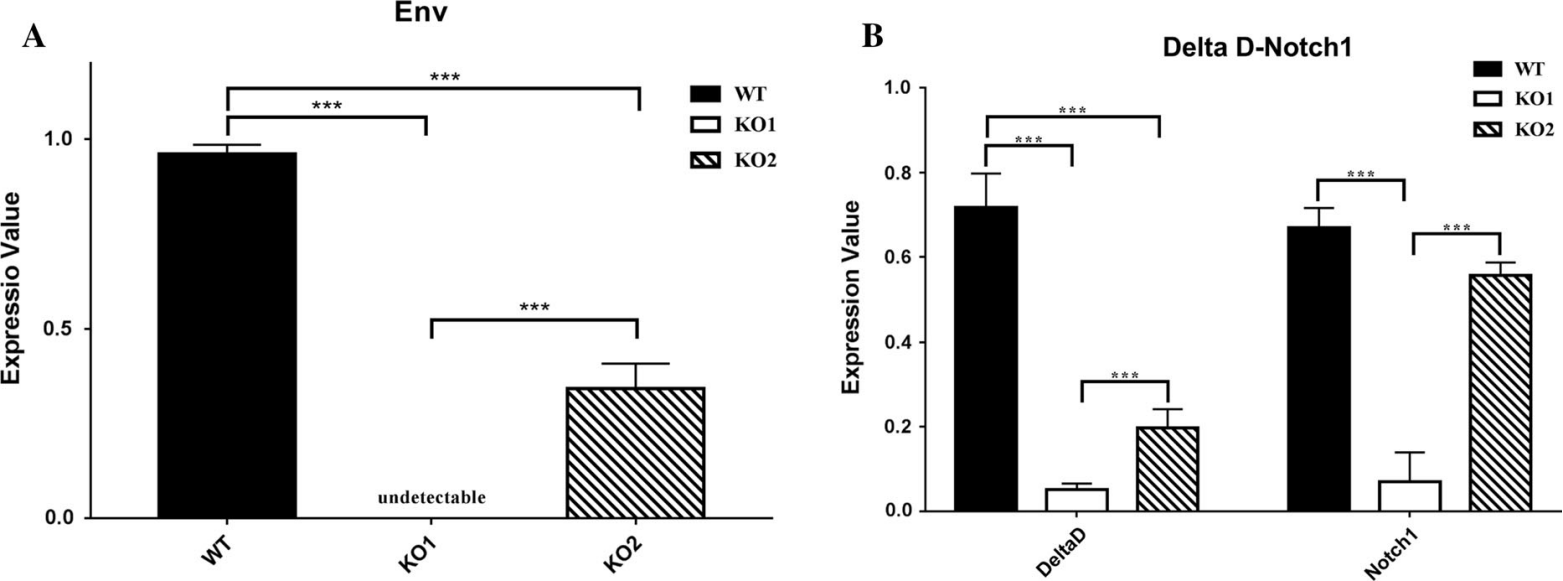
Quantitative analysis of Env, Delta D, and notch1 gene expression. **a** *Env* gene expression in TU wild-type zebrafish and ERV-knockout zebrafish generated from Cas9 transgenic fish (KO1) or by microinjection of Cas9 mRNA and LTR sgRNAs into fertilized eggs (KO2). The expression level is normalized to that in TU wild-type zebrafish. ***p < 0.001. **b** *Delta D* and *Notch1* gene expression in TU wild-type zebrafish and ERV-knockout zebrafish generated from Cas9-transgenic fish (KO1) or by microinjection of Cas9 mRNA and LTR sgRNAs into fertilized eggs (KO2). The expression level is normalized to that in TU wild-type zebrafish (Values are adjusted mean ± SEM, n = 3 for WT, n = 5 for ZFERV KO). ***p < 0.001

To exclude possible toxic effects of constitutive transgenic Cas9 expression on spinal development, fertilized wild-type zebrafish eggs were injected with Cas9 mRNA and sgRNAs targeted to ERV. As expected, embryos that developed from these eggs also showed spinal abnormalities, though the abnormal embryos constituted only approximately 30% of total embryos. Correspondingly, Env gene expression in the abnormal embryos was reduced by approximately 60% compared with wild-type embryos (Fig. 5a).

### Delta/notch signaling may mediate the role of ERV in spine development

To understand how ERV deletion causes spine abnormality, quantitative PCR analysis was performed to assess the expression of the Delta D and Notch1 genes using RNA isolated from ERV-deficient zebrafish with abnormal spines. *Delta D* and *Notch1* expression was significantly lower in zebrafish with abnormal spines than that in wild-type zebrafish (Fig. 5b), suggesting that ZFERV may be associated with spinal development and that the Delta/Notch signaling pathway may mediate the role of ZFERV. The detection of Her1, which is associated with spinal development in the notch1 signaling pathway is also shown in Supplemental Fig. 7. Her1 expression was significantly reduced in ZFERV knockout samples.

## Conclusion

In summary, a Cas9 transgenic zebrafish line was successfully established and was employed in establishing an ERV-deficient zebrafish model. Spinal abnormality is a major phenotype in ERV-deficient zebrafish, which is reported for the first time in this study. This phenotype may be attributed to the suppression of Delta D and Notch 1 gene expression due to ZFERV deletion, suggesting that the role of the ERV in spinal development may be mediated by *Delta*/*Notch* signaling.

## Electronic supplementary material

Below is the link to the electronic supplementary material.

Supplementary material 1 (DOCX 1002 kb)

## Abbreviations

Cas9: CRISPR-associated protein 9
CRISPR: Clustered Regularly Interspaced Short Palindromic Repeats
Eef1g: Eukaryotic translation elongation factor 1 gamma
Ef1α: Elongation factor 1
eGFP: Enhanced green fluorescent protein
Env: Envelop
ERV: Endogenous retrovirus
HR: Homologous recombination arm
LTR: Long terminal repeat
Mitfα: Microphthalmia-associated transcription factor α
NLS: Nuclear localization signal
RT-PCR: Reverse transcription PCR
sgRNA: Single guide RNA
Tyr: Tyrosinase
ZFERV: Zebrafish endogenous retrovirus
