# Supplementary material for: Generation of Cas9 transgenic zebrafish and their application in establishing an ERV-deficient animal model
Source: Biotechnol Lett. 2018 Sep 22;40(11):1507–18. doi: 10.1007/s10529-018-2605-5 (PMC6223727; doi:10.1007/s10529-018-2605-5)
Supplement: Supplementary file 1 — Supplementary material 1 (DOCX 1002 kb) [file 10529_2018_2605_MOESM1_ESM.docx]

**Supplemental materials**

**Supplemental Table 1. Oligoes used in this article**

| Name | Sequence (5’-3’) |
| --- | --- |
| *Ef1α*-F | CTGCAGATTTGGTAGATATCCATCAGTTCTAATG |
| *Ef1α*-R | GATTGATAAGTTTCTGCGGACCAAGATAAATGT |
| *Ef1α*-MluI-F | CGACGCGTCTGCAGATTTGGTAGATATCCATCAGTTCTAATG |
| *Ef1α*-NheI-R | CTAGCTAGCGATTGATAAGTTTCTGCGGACCAAGATAAATGT |
| *Eef1g*-F | TCATGTTATTGACACCACTACCGAT |
| *Eef1g*-R | GACGAGAGAAAGGAAGAACGAGC |
| *Eef1g*-MluI-F | CGACGCGTTCATGTTATTGACACCACTACCGAT |
| *Eef1g*-NheI-R | CTAGCTAGCGACGAGAGAAAGGAAGAACGAGC |
| eGFP-BamHI-F | CGGGATCCATGGTGAGCAAGGGCGAGG |
| eGFP-XhoI-R | CCGCTCGAGTTACTTGTACAGCTCGTCCATGCC |
| Cas9 cassette-F | CTGAGCCCACAGGCCAGCACAGGGCCCGGCCCCAGCCAGCGCTGGTTTAGTGAACCGTCAGA |
| Cas9 cassette-R | TGAGAAGGGCCATAGGGCTGTTGGGAGCGCTGGCTCCGGGACGCCAAGCTCTAGCTAGAG |
| *Mitfα*-sgRNA-F | TAATACGACTCACTATAGGCGCCGAGCACGGCATGACCCGTTTTAGAGCTAGAAATAGC |
| *Tyr*-sgRNA-F | TAATACGACTCACTATAGGAGAAAACGGTCGCTTGATGCGTTTTAGAGCTAGAAATAGC |
| LTR-sgRNA-F | TAATACGACTCACTATAGGGCGTATCTCAGTCTGTGTAGGTTTTAGAGCTAGAAATAGC |
| sgRNA-R | AAAAGCACCGACTCGGTGCCACTTTTTCAAGTTGATAACGGACTAGCCTTATTTTAACTGCTATTTCTAGCTCTAAAAC |
| Cas9-test-F | CAAACGGACAGCTCGTAGAA |
| Cas9-test-R | CGTGCAGAAAGAATCGCTTTAG |
| Cas9 insertion-test-F | AAACAGAAATTACACTTGCAAATGGAGAG |
| Cas9 insertion-test-R | CTCTTTCTCACAGTTGAGGGTG |
| *Tyr*-test-F | AACATATGTGACCCGCATCA |
| *Tyr*-test-R | TCATATTCTACTGTAATGTGAGTTTGA |
| 5’LTR-test-F | ATTGCGTTGTTTGAAATGTGAG |
| 5’LTR-test-R | CTCCAATCCATTATTTCGCTTC |
| 3’LTR-test-R | CAATATCTGCTTATAGTCCTCCTTTAACT |
| ZB Gapdh-F | CATGTTCCAGTACGACTCCAC |
| ZB Gapdh-R | CATCAATGACCAGTTTGCCG |
| ZB Actin-F | GAACCGCTGCCTCTTCTTCCTCC |
| ZB Actin-R | CCCTGTTAGACAACTACCTCCCTTT |
| ZB Ef1α-F | GGAAATTCGAGACCAGCAAATAC |
| ZB Ef1α-R | GATACCAGCCTCAAACTCACC |
| Env-F | GGGCAGAGTGGGTTTGTATTA |
| Env-R | CCATAGGAACAGCGACTCTTATC |
| Notch1-F | ACGGTGGCACAAGGGCAAA |
| Notch1-R | AGGGGTTCGGGAATTGACAT |
| DeltaD-F | AAGCACTGCACACCGACT |
| DeltaD-R | CAACCTGTAGATCTTGGGAC |
| Her1-F | GAGATCAAGGCGATTCTAGCAAGG |
| Her1-R | GGCGAGTTATGGGTTTGGATGGA |

**Supplemental Table 2. The sgRNAs used in generation of ZFERV-deficient zebrafish.**

| name | sequence |
| --- | --- |
| sgRNA-1 | TAATACGACTCACTATAGGGCAGGCTCCAAACATCAGCGTTTTAGAGCTAGAAATAGC |
| sgRNA-2 | TAATACGACTCACTATAGGCCGTATCTCAGTCTGTGTAGGTTTTAGAGCTAGAAATAGC |
| sgRNA-3 | TAATACGACTCACTATAGGAAGATGGGAAAGCGAATAGGTTTTAGAGCTAGAAATAGC |
| LTR-sgRNA | TAATACGACTCACTATAGGGCGTATCTCAGTCTGTGTAGGTTTTAGAGCTAGAAATAGC |

Red fonts denote sgRNA sequences that match to the 5’- and 3’-LTR sequences of ZFERV.

**Supplemental Table 3. The percentages of abnormal embryos resulted from injection of different sgRNAs into Cas9 transgenic zebrafish embryos**

| name of sgRNA | Percentage of abnormal embryos |
| --- | --- |
| sgRNA1 | 89.6% (448/500) |
| sgRNA2 | 84.0% (420/500) |
| sgRNA3 | 85.2% (426/500) |
| LTR-sgRNA | 86.0% (2271/2640) |

**
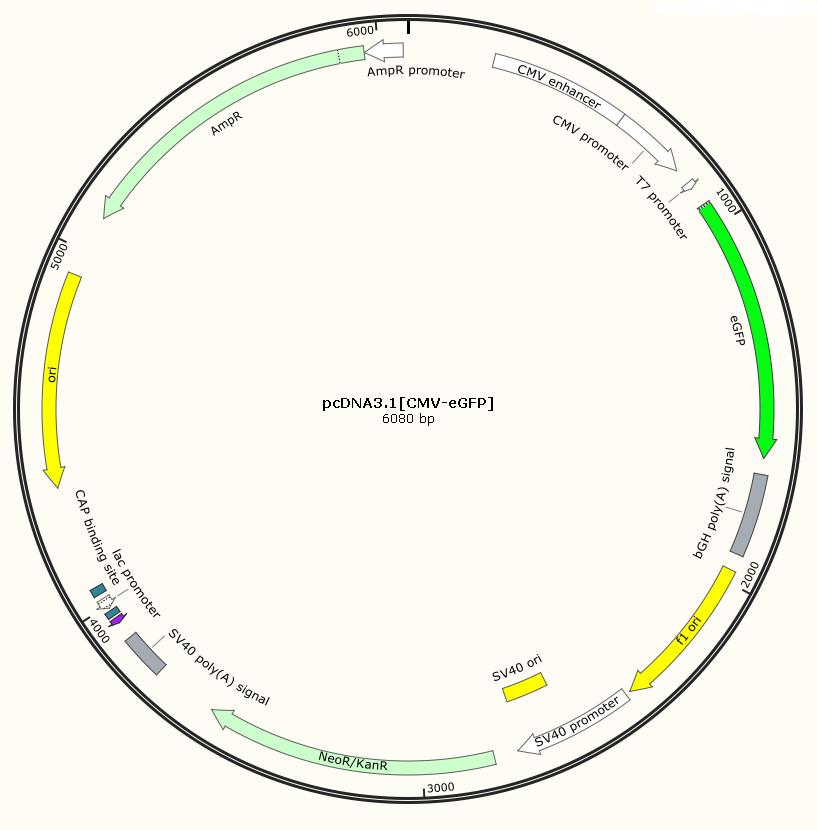
**

**Supplemental Fig. 1.** Map of pcDNA3.1[CMV-eGFP] plasmid


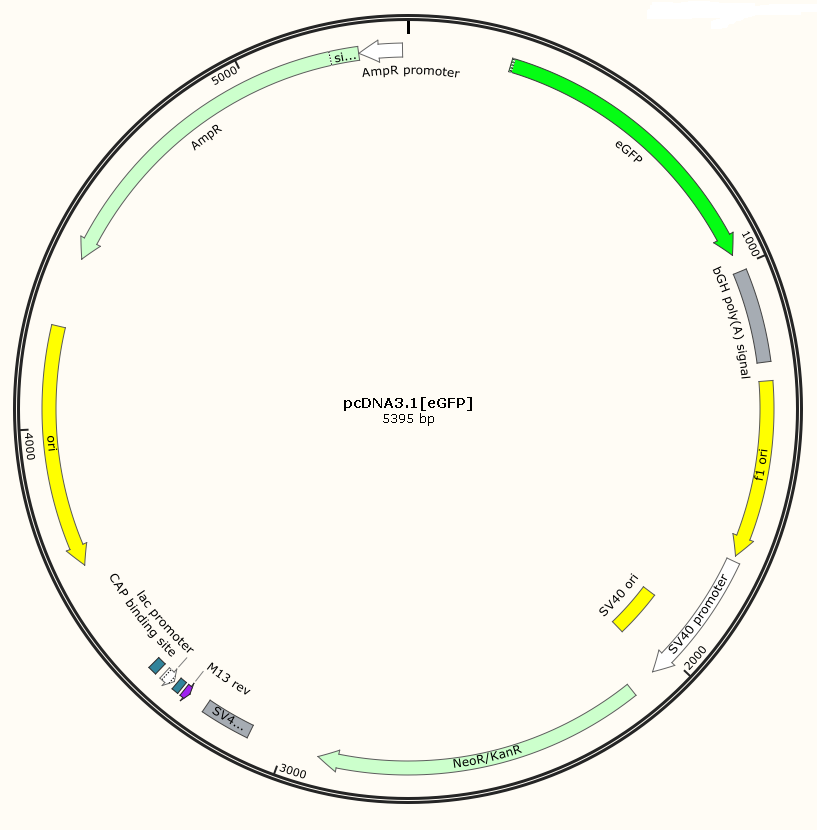


**Supplemental Fig. 2.** Map of pcDNA3.1[eGFP] plasmid


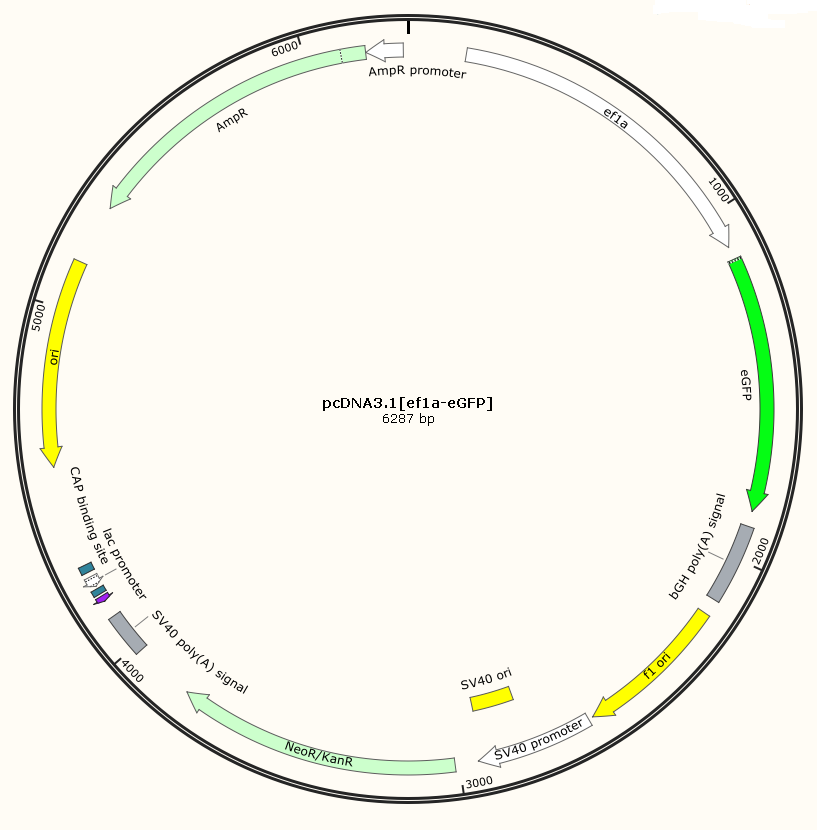


**Supplemental Fig. 3.** Map of pcDNA3.1[ef1a-eGFP] plasmid


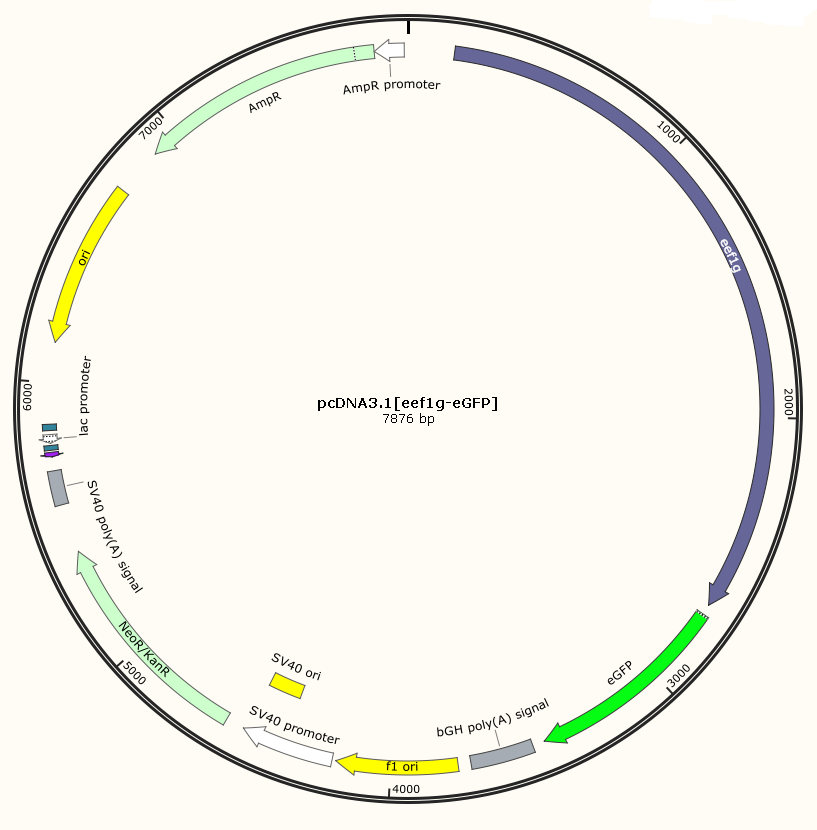


**Supplemental Fig. 4.** Map of pcDNA3.1[eef1g-eGFP] plasmid


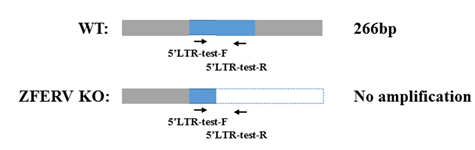


**Supplemental Fig. 5. Diagram illustrates location of the primers used to amplify 5’-LTR sequence using genomic DNA from ZFERV knockout (KO) or wild type (WT) zebrafish.** An amplicon (266 bp in size) can be produced using the primers and the genomic DNA from WT zebrafish, while no amplicons can be synthesized using the primers and the genomic DNA from ZFERV KO zebrafish. The blue box indicates the 5’-LTR sequence of ZFERV.


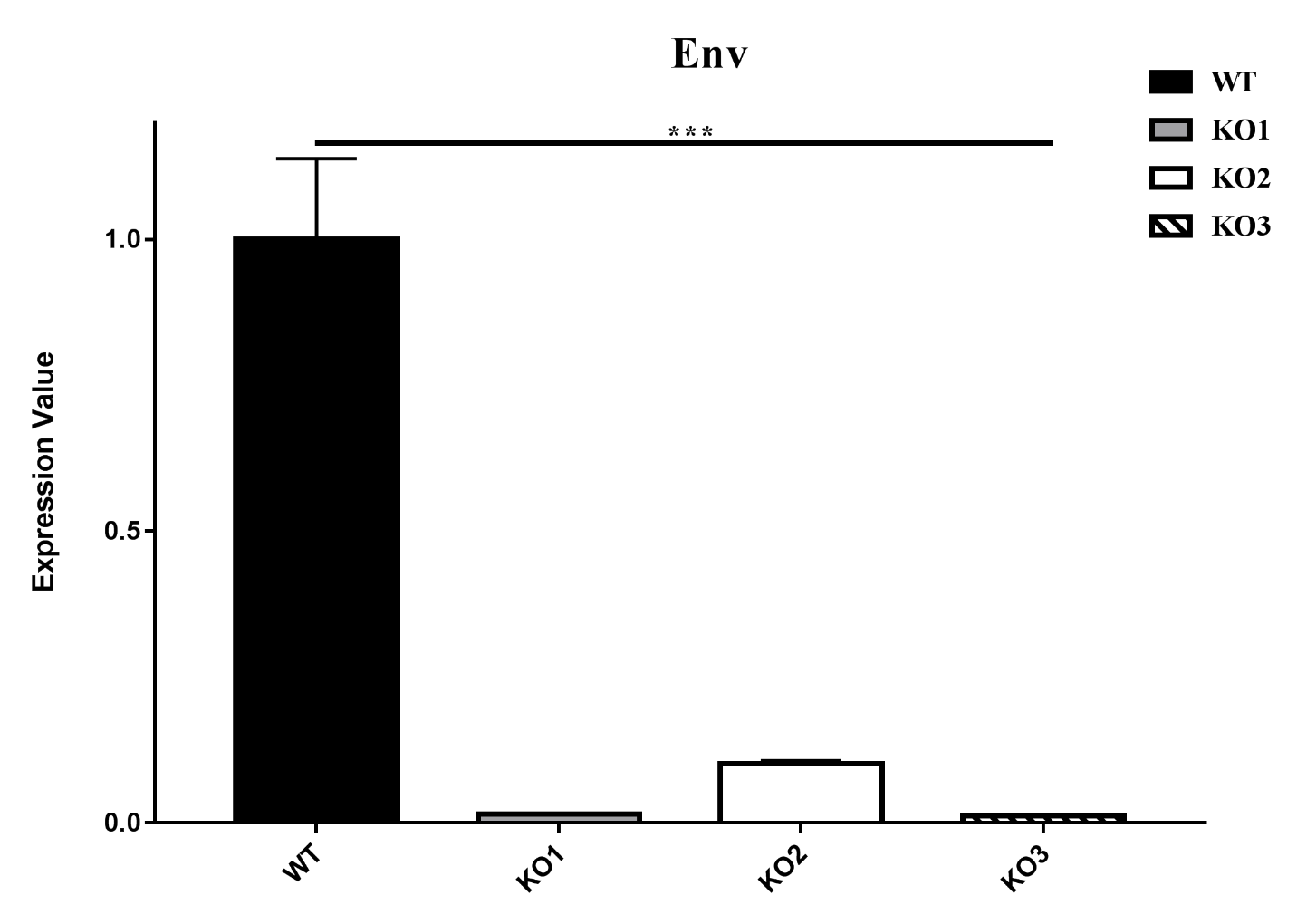
**Supplemental Fig. 6. The expression of Env gene in ZFERV knockout (KO) zebrafish embryos (KO1/KO2/KO3) resulted from injection of different sgRNAs (sgRNA1/sgRNA2/ sgRNA3) into the Cas9 transgenic zebrafish embryos vs. that in wild type (WT) embryos.** The expression of Env gene was determined by quantitative PCR with Env-specific primers. The expression of Env gene was expressed as fold change over that in WT embryos. The internal reference genes include beta-actin and GAPDH. Data are presented as mean ± SEM. n= 3 for WT, n=5 for each ZFERV-KO group. *** denotes statistical significance (p<0.001) against WT embryos.


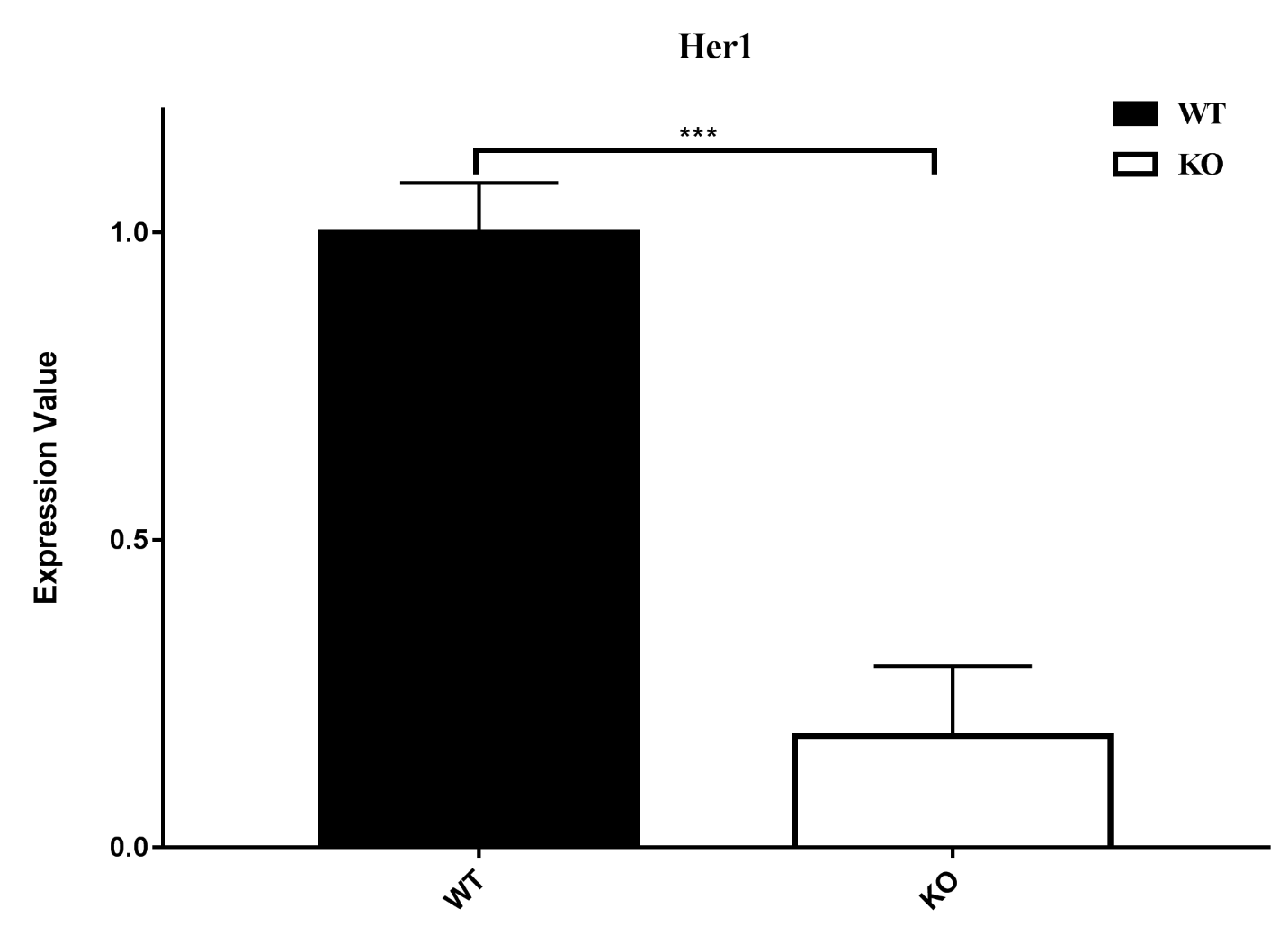


**Supplemental Fig. 7. The expression of Her1 gene in ZFERV knockout (KO) zebrafish and wildtype control (WT).** The expression of Her1 gene was determined by quantitative PCR with Her1-specific primers. The expression of Her1 gene was expressed as fold change over that in WT embryos. The internal reference genes include beta-actin and GAPDH. Data are presented as mean ± SEM. n= 3 for WT, n=5 for each ZFERV-KO group. *** denotes statistical significance (p<0.001) against WT embryos.
